# Why cognitive training is important for the health status in Parkinson’s disease: preliminary evidence from a clinical three-weeks multidisciplinary intervention

**DOI:** 10.1186/s42466-022-00210-y

**Published:** 2022-10-03

**Authors:** Jennifer Michels, Cornelius J. Werner, Beate Schumann-Werner, Jörg B. Schulz, Ana S. Costa, Kathrin Reetz

**Affiliations:** 1 Department of Neurology, Medical Faculty, RWTH Aachen University, Aachen, Germany; 2 JARA-BRAIN Institute Molecular Neuroscience and Neuroimaging, Forschungszentrum Jülich GmbH and RWTH Aachen University, 52074 Aachen, Germany; 3 Department of Neurology, Section “Interdisciplinary Geriatrics”, Department of Neurology, Medical Faculty, RWTH Aachen University, Pauwelsstraße 30, 52074 Aachen, Germany

**Keywords:** Parkinson’s disease, Cognitive impairment, Disease management, Rehabilitation, Attention

## Abstract

**Background:**

Several non-motor symptoms are present in Parkinson's disease (PD), including increasing prevalence rates of cognitive impairment during disease progression. Due to its multifaceted nature, PD management involves pharmacotherapy and non-pharmacotherapies, ideally in a multidisciplinary manner. Evidence regarding the impact of multidisciplinary interventions on motor and non-motor symptoms, as well as its impact on quality of life and daily activities of living, is limited.

**Methods:**

The aim of this real-life exploratory study was to investigate the effectiveness of a three-week clinical multidisciplinary Parkinson complex therapy (Parkinson-Komplexbehandlung, PKB), which is available as standard care for PD in the German health care system. Especially, the effect of neuropsychological attention training of 40 patients with PD was analyzed concerning their impact on motor abilities (UPDRS-III ON state), cognitive profiles and reported depressive symptoms and psychosocial function.

**Results:**

Neuropsychological data showed an improvement in response inhibition after intervention (*z* = − 2.611, *p* = 0.009). Additionally, improvements in verbal memory (*z* = − 2.318, *p* = 0.020), motor functions (UPDRS-III-score; *z* = − 5.163, *p* < 0.001) and reduction in depression symptoms (BDI-II) (*z* = − 2.944, *p* = 0.003) were also present.

**Conclusions:**

Patients with PD benefited from this multidisciplinary Parkinson complex therapy in terms of improved cognitive functioning, including attention and verbal learning, motor symptoms and emotional well-being.

## Introduction

Non-motor symptoms have been increasingly acknowledged as common features of Parkinson’s disease (PD) [1]. Cognitive impairment in PD comprises several domains and is one of the most debilitating manifestations of this slowly progressive multisystemic disease [2]. In line with this finding, we have shown differences in cognitive profiles related to the different motor phenotypes in PD [3]. PD has high prevalence rates of 40 to 80% of cognitive deficits in the course of the disease [4], with early executive deficits yielding to a more amnestic profile in the later course [5]. This is also reflected through the increasing disease burden associated with PD, which was reported as having doubled in the latest study on burden of disease of neurological disorders in Europe by Deuschl and colleagues with data of 2017 and even exceeded that of other dementias, such as Alzheimer’s disease [6].

Thus, due to the increasing health burden leading to disability in daily life and the current limited efficacy of pharmacological treatments of cognitive deficits, other therapeutic options, such as cognitive training have gained increased attention [7, 8].

Cognitive training, which comprises standardized tasks directly targeting the improvement of specific cognitive domains (e.g., attention, executive function), has shown promising effects in PD. There is evidence that overall cognition, working memory, psychomotor speed and executive functions of patients with PD can benefit from cognitive training [8]. While national guidelines (German Society for Neurology) recommend psychosocial intervention for patients with dementia [9], there is no such explicit recommendation for patients with PD [10], even though cognitive impairment is so prevalent in this patient group. Thus, as the efficacy of cognitive training is still under debate, high quality studies with randomized and controlled trials are needed [7, 11]. As multiple non-motor symptoms are usually present in PD, several studies have investigated other non-pharmacological approaches, such as physiotherapy [12, 13], occupational therapy [14], and speech and language therapy [15]. Given the multifaceted nature of PD, there are suggestions favoring the combination of non-pharmacological approaches in a multidisciplinary manner. Although only a few studies have examined multidisciplinary inpatient interventions, available evidence confirms the effectiveness of multidisciplinary therapy for the improvement of motor and non-motor abilities [16, 17]. Besides enhancing functional abilities, a combined motor and cognitive treatment seems to result in changes in brain activation patterns, which in turn could reflect a slowing down of deterioration in PD-patients [18]. Interestingly, long-term effects were also observed 12-months following such type of interventions [19]. Research found that in comparison to medical care by a general neurologist alone, individually tailored multidisciplinary therapy can improve not only motor symptoms but also quality of life (QoL) and emotional well-being [20].

In Germany, there is a specific inpatient multidisciplinary complex therapy program available as standard care for PD (Parkinsonkomplexbehandlung; PKB), which is determined by the health insurance companies, from both public and private systems. A considerable increase in PD-prevalence and an increase in annual healthcare utilization in Germany has been noticed in the past years [21]. Even though PKB has been offered as a therapeutic option in more than 200 hospitals, there is still little insight about which patients profit most from this intervention program [17].

The basis for this standardized therapy program builds on a multidisciplinary team, involving different professionals such as neurologists, physiotherapists, occupational therapists, speech therapists, as well as neuropsychologists [17]. Most of the patients undergo treatment for 14 days and studies already have shown positive outcomes on motor and nonmotor symptoms [22, 23]. Still, there are no standardized guidelines for intervention procedures.

Despite available evidence of intervention effectiveness, to our knowledge, this study is the first to investigate the effects of the multidisciplinary complex therapy program on cognition in combination with motor symptoms and QoL. The main aim of this work was to contribute to the knowledge on the effectiveness of inpatient multidisciplinary short-term complex therapy and thus to provide a starting point for a controlled study on the effectiveness of an inpatient multidisciplinary short-term complex therapy (PKB) for patients with PD. We provided additional data about a three-week multidisciplinary intervention period as well identify characteristics of patients, who would benefit from this intervention program. Specifically, the focus was on the effect of a tailored neuropsychological attention training program on attentional performance, as well as non-cognitive outcomes, such as QoL, which is recommended by the MDS Task Force [24]. In this clinical setting, we anticipated that patients show improvements in attentional performance and health status after intervention.

## Methods

Patients that underwent an inpatient stay at the Department of Neurology of the RWTH Aachen University Hospital, within the framework of a special multidisciplinary intervention (PKB), were retrospectively identified (OPS 8-97d.0–2, www.dimdi.de). The main referral requirement for PKB is the necessity for an inpatient hospital stay to undertake diagnostic measures and/or gradual optimization of pharmacological and non-pharmacological therapy, when out-patient management is not feasible. Within the multidisciplinary team lead by neurologists (specialist medical treatment management), patients typically receive at least 7.5 h therapy sessions per week, including 5 h of individual therapy, in several disciplines – neuropsychology, physiotherapy, occupational therapy, speech therapy, as well as art and music therapy. The aim of the multidisciplinary approaches is on the one hand to improve symptoms, and on the other hand, assist in autonomy maintenance and improvement in daily functioning and in social roles. Typically, medication dosing adjustments or changes also may occur during this period. In general, inpatient treatment duration ranges from at least 7 up to a total of 21 days.

The group of patients we report on had an inpatient stay of three weeks and received approximately 8 h therapy per week (for details of the various therapies, see Table 1).

**Table 1:** Actors and goals of Parkinson's complex therapy (PKB)

Actor	Goal	Average minutes of therapy as specified by OPS 8–97
Neurologist	Symptom reduction, pharmacological adjustment	–
Physiotherapist	Improving gait, balance, transfers, strength and endurance	~ 100 min
Occupational therapist	Improvement of everyday functions, maintenance of social roles, strengthening of autonomy, compensation for limitations	~ 80 min
Speech therapist	Improvement of speech function, communication and swallowing	~ 80 min
Neuropsychologist	Improvement of cognitive, affective and psychosocial functions	~ 90 min
Care	Coping with illness, counseling, general care	–
Social worker	Organization of follow-up supply, legal advice	–
Art and/or music therapy	Improvement of positive emotions, contributes to activation	group therapy; both ~ 60 min
Other disciplines	Urology, gastroenterology, nutritional advice if required	–

### Study cohort

Data was collected retrospectively from available standard clinical hospital records. In total, data of (*n* = 189) patients with PD were screened. The first assessment of this cohort was 16/08/2016, and the last patient was assessed on 10/03/2020. Out of that, data of forty (*n* = 40) patients with PD with pre-post measurements were investigated. For the remaining patients in the cohort (*n* = 149), post-intervention neuropsychological data were not available, as the neuropsychological data set was very extensive. Additionally, of the forty patients we identified afterwards responders and non-responders of intervention based on improvement/decline in at least two or more cognitive domains in inter-individual reliable indices.

Inclusion criteria for the study population included an age between 45 and 85 years and a diagnosis of idiopathic PD according to the UK Parkinson’s Disease Society Brain Bank clinical diagnostic criteria. Exclusion criteria were a diagnosis of atypical PD or PD caused by intoxication as well as a Hoehn & Yahr state of five. Furthermore, patients hospitalized for less than three weeks were also excluded.

### Pre- and post-intervention assessments

All patients were examined with an extensive standardized protocol including clinical ratings, neuropsychological tests, and patient reported outcomes measures (PROMs), which was assembled to include all major cognitive domains using standardized and commonly used tests in diagnostic and research settings. All neuropsychological assessments were carried out by trained neuropsychologists at the beginning and end of the inpatient program. The tests were scored according to standardized methods described in the published manuals. Because of the fixed clinical therapeutic procedures of PKB, no changes in clinical assessments measures were possible, and no control group could be considered for comparison with the intervention group. The comprehensive neuropsychological test battery included two screening tests, the Montreal Cognitive Assessment (MoCA) [25, 26] and Mini Mental State Examination (MMSE) [27], as well as a set of standardized tests for the assessment of the specific cognitive domains of *attention* (Test of Attentional Performance (TAP) [28], Trail-Making-Test version A (TMT-A) [29]); *memory* (Wechsler Memory Scale-Revised (WMS-R) [30] Digit span forward, Verbal Learning Memory Test (VLMT) [31] total learning, interference, immediate recall, delayed recall, and recognition, Medical College of Georgia Complex Figures (MCGCF) [32] figure delayed recall); *visuospatial functions* (MCGCF figure drawing, CORSI block tapping [33]); *language* (Consortium to Establish a Registry for Alzheimer’s Disease-Plus (CERAD +) [34] Boston Naming Test); and *executive functions* (WMS-R Digit span backward, Regensburger Wortflüssigkeitstest (RWT) [35] semantic and formal-lexical word fluency, Trail-Making-Test version B (TMT-B), Stroop [36] Word Reading, Color Naming, Interference). To avoid practice effects, alternate versions of the neuropsychological tests were used for most instruments. The order of tests was alternated regarding which version of the tests was administered.

PROMs included the Beck’s Depression Inventory (BDI-II) [37] and the SCales for Outcomes in PArkinson's disease—PsychoSocial questionnaire (SCOPA-PS) [38].

### Cognitive training

Individualized in-person cognitive training sessions lasted 30 min per session (3–4 per week), following previous studies on the effectiveness of cognitive interventions [8, 39]. Cognitive training was done with CogniPlus [40], a digital cognitive training program which includes several modalities for training attention (alertness, divided, focused, selective, visuo-spatial, neglect, visual field, vigilance). CogniPlus adapts dynamically to the level of difficulty based on the patients’ ability. At the end of each training session numerical and graphical feedback can be obtained.

### Statistical analyses

Neuropsychological tests were analyzed prior and after intervention to determine the impact of the intervention program on cognitive performance and on the health status. Differences over time (pre-/post-intervention) were tested for the group and intra-individual levels. As normality assumption was not fulfilled for more than 85% of the neuropsychological tests (Shapiro–Wilk W Test *p* < 0.05), nonparametric tests were chosen. Tests were two-sided; the alpha level was set to 0.05.

The Wilcoxon-signed-rank test was used to compare pre- and post-intervention performance at the group level with standard normal distributed z-value to test for significance. Spearman’s correlation coefficients were used to assess test–retest reliability. Because of the use of non-parametric tests and the small sample size, unbiased Cohen’s d values were generated in order to calculate effect sizes values of practice effects. Subsequently, an individual score of change was calculated for each patient to avoid measurement error and practice effects. Afterwards, a previously developed reliable change index (RCI) methodology was operated. This RCI-method, following Tröster et al*.* [41], was defined as ((T2 − T1) − (M2 − M1))/SD, with T1 representing the observed raw score before intervention, T2 representing the observed raw score after intervention, SD representing the standard deviation of the test–retest difference for the whole group, M1 representing the group mean test score before intervention, and M2 representing the group mean test score after intervention. For correction of practice effects, the average group change constant was also considered [42]. For reliable change, the alpha was set to 0.10 (two-tailed) with a reliable improvement occurring when scores are higher than + 1.645, and a decline when values exceed − 1.645, except for time-based tasks which need to be interpreted the other way around.

Finally, based on the prior determined reliable change, patients were classified as responders or non-responders, with improvement/decline in at least two or more cognitive domains. Inter-individual differences between these two groups were calculated using the Mann–Whitney nonparametric test.

Statistical data analyses were carried out using SPSS version 24 (IBM).

## Results

### Clinical characteristics and medication

Patients were on average 68.3 years old (*SD* = 8.51), had on average 13.3 years of education (*SD* = 2.74) and an average Hoehn & Yahr stage of 2.8 (*SD* = 0.67). Two third of patients were male and patients had a disease duration of approximately nine years (*M* = 108.67 months, *SD* = 74.76). At the time of admission, all patients were taking PD-specific medication (levodopa and/or dopamine agonists). Levodopa equivalence daily dose (LEDD) [43] increased from 713 mg at admission to 843 mg at discharge. Furthermore, before intervention seven patients were taking dementia-specific medication (cholinesterase inhibitors), twenty-one patients were taking antidepressants and four patients were taking neuroleptics (see Table 2). During intervention, medication of antidepressants and dementia-specific medication remained stable. Only one patient started dementia-specific medication (rivastigmine) during the intervention period. Patients already taking dementia-specific drugs had worse scores on cognitive tests (Kruskal Wallis test, *p* < 0.05), for example, in MoCA and MMSE or tests measuring memory functions, compared to patients without. Moreover, patients with previous antidepressant treatment had lower BDI-scores compared to patients not taking antidepressant medication (Kruskal Wallis test, *p* < 0.05).

**Table 2:** Demographics, clinical characteristics, test–retest reliability, practice effects, and reliable change indices

	*n*	Before Intervention	*n*	After Intervention	*d*	*r*	*M* Diff (S.D.)	90% CI
Clinical characteristics								
Age (years)	40	68.32 ± 8.51		–	–	–	–	–
Male (%)	27	67.5		–	–	–	–	–
Education (years)	40	13.30 ± 2.74		–	–	–	–	–
Disease duration (months)	40	108.67 ± 74.76		–	–	–	–	–
Hoehn & Yahr stage	37	2.78 ± 0.67		–	–	–	–	–
UPDRS III ON state	38	28.68 ± 12.47	35	20.97 ± 10.71	0.66	0,82**	7.71 (11.62)	6.89, 10.65
*Medication*								
*Parkinson medication*								
LEDD (mg/day)	40	713.65 ± 335.82	40	843.57 ± 437.21	− 0.33	0,79**	129.9 (389.83)	− 192.29, − 67.55
NMDA antagonists	8	-	11	**–**	**–**	**–**	**–**	**–**
L-Dopa	37	-	37	**–**	**–**	**–**	**–**	**–**
Dopamine agonists	24	-	28	**–**	**–**	**–**	**–**	**–**
COMT-inhibitor	5	-	19	**–**	**–**	**–**	**–**	**–**
MAO-β-inhibitor	6	-	5	**–**	**–**	**–**	**–**	**–**
*Dementia-specific drugs*								
Cholinesterase inhibitor	7	**-**	8	**–**	**–**	**–**	**–**	**–**
*Antidepressants*								
SSRIs	8	**–**	8	**–**	**–**	**–**	**–**	**–**
SNRIs	7	**-**	6	**–**	**–**	**–**	**–**	**–**
NaSSA	6	**-**	6	**–**	**–**	**–**	**–**	**–**
Neuroleptics	3	**-**	3	**–**	**–**	**–**	**–**	**–**
*Neuropsychological variables*								
*Screening Tests*								
MMSE (total)	40	27.53 ± 2.35	40	27.58 ± 2.83	− 0.02	0.65**	− 0.05 (2.60)	− 0.57, 0.47
MoCA (total)	40	24.00 ± 4.06	40	23.63 ± 3.35	0.10	0.69**	0.37 (3.72)	− 0.48, 1.18
*Emotional well-being/ Psychosocial Scales*								
BDI-II (total)	40	12.33 ± 7.39	40	10.30 ± 6.89	0.28	0.79**	2.03 (7.15)	0.74, 3.31
SCOPA-PS (total)	40	10.53 ± 5.85	40	10.23 ± 5.77	0.05	0.75**	0.3 (5.81)	− 0.77, 1.37
*Memory*								
Word list direct recall (VLMT)	40	4.28 ± 1.85	40	6.18 ± 6.90	− 0.38	0.35**	− 1.9 (5.05)	− 3.80, 0.01
Word list learning (VLMT)	40	38.18 ± 11.12	40	39.73 ± 11.23	− 0.14	0.76**	− 1.55 (11.18)	− 3.60, 0.50
Word list delayed recall (VLMT)	40	6.73 ± 3.97	40	6.70 ± 3.90	0.01	0.62**	0.03 (3.94)	− 0.87, 0.92
Word list recognition (VLMT)	40	7.98 ± 5.32	40	6.25 ± 5.82	0.31	0.66**	1.73 (5.58)	0.37, 3.08
Word list delayed recall—Intrusions (VLMT)	40	6.63 ± 4.90	40	7.10 ± 5.07	− 0.09	0.53**	− 0.47 (4.99)	− 1.75, 0.80
Word list delayed recall—errors (VLMT)	40	5.10 ± 4.35	40	5.53 ± 5.19	− 0.09	0.67**	− 0.43 (4.79)	− 1.46, 0.61
Figure delayed recall (MCGCF)	40	15.65 ± 9.11	40	16.95 ± 9.83	− 0.14	0.63**	− 1.3 (9.48)	− 3.40, 0.80
Digit span forward (WMS-R forward)	40	6.60 ± 1.65	40	6.93 ± 1.23	− 0.23	0.27	− 0.33(1.46)	− 0.77, 0.12
*Executive*	40							
Semantic word fluency (RWT)	40	15.05 ± 6.77	40	16.38 ± 6.08	− 0.21	0.35**	− 1.33 (6.43)	− 3.30, 0.65
Formal-lexical word fluency (RWT)	40	26.20 ± 8.88	40	27.15 ± 9.99	− 0.10	0.79**	− 0.95 (9.45)	− 2.47, 0.57
Digit span backward (WMS-R backward):	40	5.28 ± 1.77	40	5.13 ± 1.40	0.09	0.57**	0.15 (1.56)	− 0.26, 0.56
TMT-B Sec	38	166.29 ± 81.10	40	160.13 ± 80.64	0.08	0.77**	6.16 (80.86)	− 3.56, 25.51
*Attention*								
Stroop Word Reading	40	40.07 ± 11.00	40	39.75 ± 10.31	0.03	0.93**	0.32 (10.66)	− 0.95, 1.60
Stroop Color Naming	40	62.05 ± 20.40	40	59.22 ± 16.01	− 0.15	0.86**	2.83 (18.34)	− 0.76, 6.41
Stroop Interference	38	119.76 ± 36.47	38	121.87 ± 55.21	− 1.12	0.79**	− 2.11 (1.88)	− 3.70, 19.51
TMT-A Sec	40	57.08 ± 27.51	40	54.70 ± 27.26	0.09	0.78**	2.38 (27.39)	− 2.05, 6.80
TAP-Alertness (RT ms Intrinsic)	40	338.83 ± 74.65	40	333.28 ± 96.30	0.06	0.73**	5.55 (86.16)	− 11.51, 22.61
TAP-Alertness (RT ms Phasic)	40	336.95 ± 129.78	40	312.78 ± 87.45	0.22	0.66**	24.17 (110.66)	− 1.30, 49.65
TAP-GoNoGo RT ms	40	464.05 ± 91.81	40	430.22 ± 58.61	0.44	0.55**	33.83 (77.02)	13.35, 54.30
TAP-GoNoGo—errors	40	3.35 ± 3.11	40	3.78 ± 2.89	− 0.14	0.49**	− 0.43 (3.00)	− 1.32, 0.47
TAP-GoNoGo—omissions	40	1.23 ± 1.97	40	1.00 ± 1.81	0.12	0.42**	0.23 (1.89)	− 0.28, 0.73
TAP-Divided Attention – visual (RT ms)	30	1004.00 ± 186.38	19	981.00 ± 198.45	0.12	0.54*	23 (191.09)	− 29.37, 108.14
TAP-Divided Attention – visual (errors)	30	3.13 ± 3.64	19	1.79 ± 2.12	0.45	0.27	1.34 (2.98)	0.08, 2.58
TAP-Divided Attention – visual (omissions)	30	3.10 ± 2.78	19	3.79 ± 2.76	− 0.25	0.56*	− 0.69 (2.77)	− 1.49, 1.05
TAP-Divided Attention – auditory (RT ms)	29	607.41 ± 117.91	18	590.56 ± 110.80	0.15	0.65**	16.85 (114.41)	− 40.12, 52.83
TAP-Divided Attention – auditory (errors)	30	6.03 ± 5.33	20	5.85 ± 8.35	0.03	0.62	0.18 (7.00)	− 4.47, 3.83
TAP-Divided Attention – auditory (omissions)	30	0.73 ± 0.94	20	1.85 ± 4.74	− 0.33	0.76**	− 1.12 (3.42)	− 2.86, 0.65
TAP-Divided Attention – double condition – auditory (RT ms)	34	673.74 ± 159.82	35	626.29 ± 140.18	0.32	0.55**	47.45 (150.32)	− 4.75, 98.19
TAP-Divided Attention – double condition – visual (RT ms)	35	951.46 ± 208.26	37	918.84 ± 147.38	0.18	0.38*	32.62 (180.41)	− 25.18, 104.60
TAP-Divided Attention – Double condition (errors)	35	9.11 ± 8.59	37	5.49 ± 6.62	0.47	0.43*	3.62 (7.67)	1.55, 5.57
TAP-Divided Attention – Double condition (omissions)	35	5.17 ± 5.32	37	4.73 ± 4.28	0.09	0.65**	0.44 (4.83)	− 0.42, 1.48
*Visuospatial*								
Block Tapping (CORSI)	40	4.80 ± 1.02	40	4.83 ± 0.84	− 0.03	0.56**	− 0.03 (0.93)	− 0.26, 0.21
Figure copying (MCGCF)	40	32.86 ± 6.36	40	33.23 ± 5.07	− 0.06	0.64**	− 0.37 (5.75)	− 1.28, 0.56
*Language*								
BNT (CERAD +)	40	14.25 ± 1.57	40	14.50 ± 1.26	− 0.18	0.62**	− 0.25 (1.42)	− 0.53, 0.03

Data are given as mean ± SD if not indicated otherwise. Percentages refer to the number of patients in each column if not indicated otherwise. Neuropsychological data show raw scores if not indicated otherwise. Spearman’s rho correlation coefficients were used to assess test–retest reliability

*BDI-II* Beck’s depression inventory, *BNT* Boston naming test, *CERAD +*, Consortium to establish a registry for Alzheimer’s disease-plus, *LEDD* Levodopa equivalent daily dose, *MCGCF* Medical college of georgia complex figures, *MMSE* Mini mental state examination, *MoCA* Montreal cognitive assessment, *NaSSA* Noradrenergic and specific serotonergic antidepressants, *PD* Parkinson’s disease, *PKB* Parkinsonkomplexbehandlung, *RT* Reaction time, *RWT* Regensburger Wortflüssigkeitstest, *SCOPA-PS* SCales for outcomes in PArkinson's disease—PsychoSocial questionnaire; *SNRI* Serotonin-noradrenaline reuptake inhibitors, *SSRI* Selective serotonin reuptake inhibitors, *TAP* Test of attentional performance, *TMT-A* Trail making test version A, *TMT-B* Trail making test version B, *UPDRS* Unified Parkinson’s disease rating scale, *VLMT* Verbaler Lern- und Merkfähigkeitstest, *WMS-R* Wechsler memory scale-revised

Medication: Dopamine agonists: Piripedil, Pramipexol, Ropinirol, Rotigotin, Apomorphin, Cabergolin, Pergolid, L-Dopa: controlled Levodopa, immediate Levodopa, Stalevo, MAO-β-inhibitor: Selegilin, Rasagilin., NMDA antagonists: Amantadin, Budipin, COMT-inhibitor: Entacapon, Tolcapon, Acetycholinergics: Biperiden, Cholinesterase inhibitors: Rivastigmine, Galantamine, SSRI: Sertralin, Citalopram, Escitalopram, SNRI: Duloxetin, Venlafaxin; NaSSA: Mirtazapin, Neuroleptics: Clozapin, Quetiapin

*d*, Unbiased Cohen’s d, *r*, Spearman’s rho correlation coefficient, *M*, mean, *Diff*, difference score, *S.D*., standard deviation, *CI*, confidence interval

^*^ *p* < 0.05

^**^ *p* < 0.01

^***^ *p* < 0.001

Overall, the three-week treatment led to a clinical improvement in motor abilities measured by the UPDRS-III obtained in the ON state (*pre*: *M* = 28.68, *SD* = 12.47; *post*: *M* = 20.97, *SD* = 10.71).

### Clinical and cognitive parameters after intervention

The cognitive status at baseline was determined with the MoCA cut off raw score. Based on Dalrymple-Alford and colleagues (2010), the optimal MoCA cut-off score for mild cognitive impairment (PD-MCI) is < 26 and the optimal MoCA cut-off score for dementia (PD-D) is < 21 [44]. Thus, *n* = 15 patients had a normal cognitive profile, *n* = 15 were classified as PD-MCI and *n* = 10 as PD-D.

Differences in cognition after intervention for the cohort are displayed in Table 2. As shown, we found considerable variability of test performances before and after intervention, suggesting that some PD-patients do in fact show cognitive improvements and declines that are covered by the group-level statistics.

Using Wilcoxon-signed-rank test for the cognitive parameters over time at the group level, there was a clear reduction in reaction times in response inhibition in TAP-GoNoGo task (*z* = -2.611, *p* = 0.009). Furthermore, the number of errors in the TAP divided attention double condition decreased, showing that patients responses were more accurate after training (*z* = 2.806, *p* = 0.005). Additionally, we found a clear improvement in verbal learning, showing that after intervention PD-patients could retain more words at the first trial of a wordlist task (*z* = − 2.318, *p* = 0.020). Moreover, we found an improvement in motor functions (UPDRS III-score; *z* = − 5.163, *p* < 0.001) and in depressive symptoms (BDI-II) after intervention (*z* = − 2.944, *p* = 0.003), however, no differences were found for self-report psychosocial functioning, as measured by the SCOPA-PS.

After correction for multiple comparisons the applied alpha of 0.001, however, showed no significant results.

### Intraindividual improvements

**Fig. 1:**
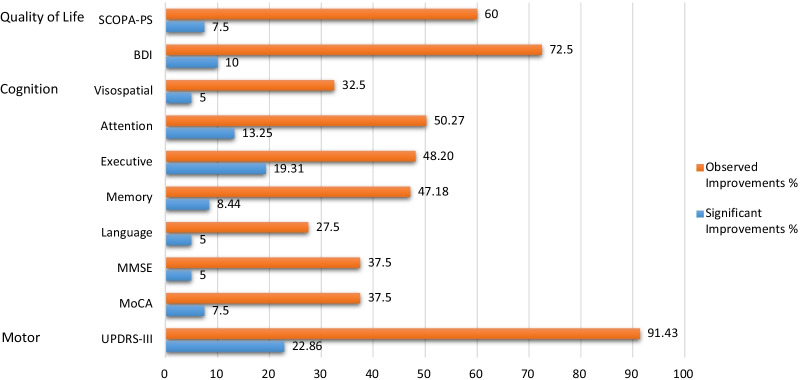
Percentage of PD-patients (total) showing observed and significant improvements in cognitive performance after intervention compared to performance before. “Observed improvements” are seen in patients showing improvements in raw scores, indicating a trend, which, however, cannot be obtained statistically. “Significant improvements” are seen in patients who did show improvements calculated using the RCI method. Most patients showed unchanged cognitive performance (z score ≥ 1.645 to ≤ 1.645). A low proportion of patients showed non-significant improvements (z score ≤ 1.645) after intervention; The percentage of patients presenting with significant improvement (z score ≥ 1.645) after intervention was observed mainly in motor function and time-dependent tasks related to attention and executive functions. *BDI*-II, Beck’s depression inventory; *MMSE* Mini mental state examination, *MoCA* Montreal cognitive assessment; *SCOPA-PS* Scales for outcomes in Parkinson's disease—Psychosocial questionnaire, *UPDRS* Unified Parkinson’s disease rating scale. Overview composites: see Table 2)

Using the RCI-method, we calculated the percentage of PD-patients showing significantly improved, stable, or not significantly improved cognitive performance after intervention. Although raw scores improved, intraindividual statistics suggest that most patients showed unchanged cognitive performance (z scores ≥ 1.645 to ≤ 1.645), considering their own performance before intervention and practice effects (see Fig. 1). Compared to pre-intervention, eight patients (22.86%) present a significant improvement in motor function (UPDRS III), while the majority (24 patients, 68,57%) remained stable, and only for three patients (8.57%) a decrease was present. A similar pattern emerged for executive functions and attentional performance.

In general, in 16.65% of PD-patients we found an improvement at the intra-individual level in reaction times in executive function tests after intervention, while the majority (65.83%) remained stable Especially time-dependent executive and attention tasks showed significant improvements. For example, in the interference condition of the Stroop test eight patients (21,62%) were faster and twenty-six patients (70.27%) remained stable. In tests measuring attention, in general 13.25% improved significantly, while 70.32% showed comparable performance after intervention.

### Effect of training on attention

As attentional functions were of prior interest, given that patients underwent specific neuropsychological attention training, we report in more detail possible changes in performance in attentional functions, measured by the TAP. Regarding intrinsic alertness, we found reliable intra-individual improvements in reaction time for seven patients (17.5%). In the phasic (cued) condition, improvement was seen in six patients (15%).

For the GoNoGo task, measuring inhibition control, seven patients (17.5%) show reliable faster reaction times. When performing the divided attention task, given its complexity, several patients were not able to complete the double condition task successfully. In the auditive part of the double condition, with data available for both time points, four patients (12.5%) showed a reliable reduction of reaction times. In the visual task within the double condition for four patients (11.76%) reliable changes could be detected.

### Responders vs. non-responders

Patients were classified as responders (*n* = 22) or non-responders (*n* = 14) of intervention based on inter-individual reliable indexes in order to investigate possible characteristics suggestive of intervention success. Males were more responsive to intervention (72.7%) and in general emotional well-being was rated to be better for responders. Education was comparable in both (*M* = 13.59 vs. *M* = 13.21), while responders were slightly older (*M* = 68.14 vs. *M* = 67.43), had a higher Hoehn & Yahr state (*M* = 2.79 vs. *M* = 2.64), lower UPDRS-III-scores (*M* = 27.38 vs. *M* = 30.23) and showed lower MoCA-scores (*M* = 23.82 vs. *M* = 24.43) compared to non-responders.

Four patients showed ambivalent changes so that they were excluded from further analyses. No demographic group differences were present in post-hoc multiple comparisons pre- and after intervention. Nevertheless, overall cognitive abilities before intervention were reduced in responders when compared to the group of non-responders. Particularly, responders were slower in timed and attention tests, which was underlined by significant inter-individual differences. Using the Mann–Whitney nonparametric test, we found no differences between responders and non-responders before intervention in cognition. After intervention, significant differences in reaction time for phasic Alertness (*U* = 84, *p* = 0.023) and GoNoGo (*U* = 87, *p* = 0.030) were present, showing that responders got faster compared to non-responders.

## Discussion

Our study provides preliminary evidence that motor and distinct cognitive abilities of PD-patients were slightly improved after three-week multidisciplinary intervention. We found both a reduction in reaction times and increase in accuracy in tests measuring attentional functions, as well as improved verbal memory retrieval. Responders benefitted exceptionally from intervention in the cognitive domain’s executive functions and attention. Finally, better ratings of emotional well-being were also found for post-intervention.

Even considering the high Hoehn & Yahr stage of this PD-cohort, improved motor scores were found after intervention, with approximately 22% showing a reliable improvement when using intraindividual reliable change methods. These results can be linked to other studies reporting enhancements in motor abilities after this type of interventions [22, 23].

Next to improvements in motor functions, improvements were found for executive functions, attention and were specifically relevant in time-dependent tasks, such as the Trail-Making-Test and the Stroop Test. Using intraindividual reliable change statistics, data indicate a reliable improvement in executive functions for 20% and in attention tests for 13% of patients with PD. It must be mentioned that neuropsychological training sessions during the intervention period had a major focus on these cognitive domains. Our results show that the specific attention training yielded in an improvement in reaction time, especially in intrinsic alertness and inhibition control, however we are aware that these effects cannot be derived solely from the CogniPlus training. Nonetheless, as far as we know, this is the first attempt to analyze the effect of attention training in patients with PD within a multidisciplinary and intensive intervention setting in an exploratory way. Our results relate to previous research showing that a multidisciplinary therapy might improve functional abilities [8, 16]. More particularly, a study by Lawson et al*.* [45] revealed that interventions which improved attention could also have an improving impact on QoL. Our study was also capable of indicating this effect by displaying enhancements in emotional well-being, as seen in previous studies [22, 23]. These results can be interpreted as positive patient capability on grounds of the sheltered hospital atmosphere and experiences of self-confidence.

After analyzing inter-individual differences and therefore classifying responders and non-responders of therapy, we found that significant differences in reaction time for alertness and response inhibition were present, indicating that non-responders were slower in processing speed and attention after multidisciplinary intervention therapy, including neuropsychological training session of attention training. Our study revealed that males were more responsive to intervention and responders seem to have a more stable emotional state in general. Responders were slightly older and had a higher Hoehn & Yahr state, however, motor abilities were less impaired compared to patients who did not benefit from intervention. Furthermore, responders’ overall cognitive abilities before intervention were reduced, with lower scores in MoCA but not MMSE compared to non-responders. This means that responders had a greater range to benefit more from the intervention, while non-responders were mainly patients with a better health condition and were therefore fitter and could not obtain significant improvement, known as ceiling effect.

All in all, this study represents a first exploratory attempt to investigate the effectiveness of a multidisciplinary intervention and we would like to underline that this is a pilot study regarding its’ primary outcome and arises from a particular clinical setting. Taken together, our data indicate a beneficial impact of combined therapies in enhancing cognitive functions such as attention and learning [46, 47]. Furthermore, our retrospective analysis also indicates that multidisciplinary therapy is an effective possibility for inpatient treatment of PD measured by improved motor scores (UPDRS-III) and lower depression scores (BDI-II) in PD.

This is especially prominent as our cohort is in an advanced stage of PD (Hoehn & Yahr *M* = 2.89), demonstrating that also seriously affected patients still can improve their state of health.

Of course, antidepressant medication have an impact on depressive symptoms. However, as antidepressant medication did not change during the intervention period, we assume, that the reductions of depressive symptomatology (BDI-II) might be due to the activation during intervention, which promote the handling of dysfunctions. Furthermore, and beyond indirect effects of motor improvement, PD-patients were supported on how to better adjust in daily living tasks, which could also have an enhancing effect on their mood.

This can be related to findings by Ellgring and colleagues, showing that PD-patients benefited from structured psychological interventions in terms of changed dysfunctional behaviors and cognitions, which in turn improved emotional distress and depression, in patients just as with their relatives [48].

The overall strength of the study is that we revealed a deeper insight into the therapeutic success of therapies in PD and provided first evidence how to improve intervention programs to foster cognitive improvement and related health state.

Besides the small sample size, which limited the statistical power in all the analyses, and therefore the ability to reveal potential significant intervention factors, the study has some further limitations given its’ primarily exploratory nature. Considering that the interventional program, has a priori defined criteria and therapeutic recommendations, there was limited flexibility regarding used clinical measures and given the fixed clinical standards of the intervention program, no control group could be considered for comparison with the intervention group. We are aware of our complex study design, and analyses focusing on single exposure of interest can never fully account for its entirety of the multidomain of this intervention. The problem in all multimodal exercise programs is, that it is impossible to account for all possible variables. It was not feasible to add all types of therapies as co-variates to outcomes. Therefore, we concentrated, on the most important factors. Also given these pre-determinants regarding the intervention program, more complex study designs including a control group could be considered in future research. Thus, a causal relation between the multidisciplinary therapy and the illustrated improvements cannot be claimed. Applying a fully standardized test could be advantageous, as there is a particular necessity for more studies testing the efficacy of cognitive training in PD [11]. One future and necessary goal should be to organize a working-group of PD-experts to establish a set of cognitive tests as well as one common and standardized set of intervention program.

Due to personnel and logistic constraints, not all tests were done by all patients and only patients who were able to participate, received a specific neuropsychological attention training. Next, all patients were taking PD-specific medication, which was adapted during inpatient admission, if applicable, and mainly in the first two weeks of intervention, so that LEDD increased. This might have an impact on cognition, as seven patients were also taking cholinesterase inhibitors (Rivastigmine). Similarly, psychtropic medication might have an impact on depression, although the dose was not changed during intervention. However, there was no significant relationship between dopaminergic medication and responders/non-responders (*X*^*2*^ (1, *N* = 36) = 0.14, *p* > 0.05.), which is in line with other real-world data. For example, a recent study by Rosenfeld, *et. al* showed that motor execution improved with medication, however the level of information processing did not with medication, which only could be achieved through exercise [49]. The multi-modal aspects of intervention, including occupational therapy, physiotherapy, speech therapy and neuropsychology, of course all can have an effect. The improvement we found is therefore probably not exclusively due to specific components of the received treatment, but might also not be solely due to optimization of medication effects. Additionally, pharmacological treatment has been shown to have only limited profit in the treatment of cognitive impairment in PD [50]. Furthermore, none of the patients or the examiners were blinded which could have biased the examiners' evaluation and patients' self-reports.

Finally, although the SCOPA-PS is recommended by the MDS, there is still some criticism that this questionnaire has a greater focus on psychosocial adjustment and is not fully assessing QoL (e.g. lack of questions concerning physical and mental health) [24]. Finally, future analysis with a bigger cohort might yield significant results.

We would like to stress that this was a first attempt to explore the effects of specific training, in our case attention training on specific attention deficits. It is important to look at this rather theoretical question prior to looking for training effects on attention performance in everyday living. Our study might help other researchers in assessing statistically meaningful alterations in cognitive performance, since not all PD-patients will show the average expected variance in cognition.

Involving different professionals into a multidisciplinary team is crucial for overcoming the complexity of PD. This can easily be transferred to other health care systems and settings worldwide.

## Conclusion

Our findings indicate the usefulness of multidisciplinary Parkinson complex therapy as motor and cognitive abilities, including attention and verbal learning, as well as emotional well-being of PD-patients were slightly improved after three-weeks of intervention. The considerable increase in PD-prevalence and in annual healthcare utilization pose a huge challenge for the health care system in Germany. Therefore, it is crucial to gain a deeper insight into the therapeutic success of therapies on disease-related changes in PD as patients might benefit from well-designed and individual therapeutic interventions. Whether the positive effect of multidisciplinary intervention on motor performance and QoL lasts for a much longer period remains to be proven, but motivating and guiding PD-patients to adjust daily living in their daily lives seems promising.

## Data Availability

The datasets used and analyzed during the current study are available from the corresponding author on reasonable request.

## Abbreviations

BDI-II: Beck’s depression inventory
BNT: Boston naming test
CERAD +: Consortium to establish a registry for Alzheimer’s disease-plus
CI: Confidence interval
D: Unbiased cohen’s d
Diff: Difference score
LEDD: Levodopa equivalent daily dose
M: Mean
MCGCF: Medical college of Georgia complex figure
MMSE: Mini mental state examination
MoCA: Montreal cognitive assessment
NaSSA: Noradrenergic and specific serotonergic antidepressants
PD: Parkinson’s disease
PKB: Parkinsonkomplexbehandlung;
R: Spearman’s rho correlation coefficient
QoL: Quality of life
RCI: Reliable change index
RT: Reaction time
RWT: Regensburger Wortflüssigkeitstest
SCOPA-PS: Scales for outcomes in Parkinson's disease—psychosocial questionnaire
SNRI: Serotonin-noradrenaline reuptake inhibitors
SSRI: Selective serotonin reuptake inhibitors
S.D.: Standard deviation
TAP: Test of attentional performance
TMT-A: Trail making test version A
TMT-B: Trail making test version B
UPDRS: Unified Parkinson’s disease rating scale
VLMT: Verbaler Lern-und Merkfähigkeitstest
WMS-R: Wechsler memory scale-revised
